# Examining ethical leadership in health care organizations and its impacts on employee work attitudes: an empirical analysis from Austria

**DOI:** 10.1108/LHS-06-2020-0034

**Published:** 2016-06-02

**Authors:** Arleta Anna Franczukowska, Eva Krczal, Christine Knapp, Martina Baumgartner

**Affiliations:** Department for Economy and Health, Danube University Krems, Krems, Austria; at-MO-sphere Immobilienverwaltung e.U, Klagenfurt/Wörthersee, Austria; Infineon Technologies Austria AG, Villach, Austria

**Keywords:** Health-care, Austria, Burnout, Job satisfaction, Affective commitment, Ethical leadership, Frustration tolerance, Emotional stability

## Abstract

**Purpose:**

This study aims to examine the effects of ethical leadership on job satisfaction, affective commitment and burnout of health care employees, considering frustration tolerance and emotional stability as moderating variables.

**Design/methodology/approach:**

A questionnaire was used to survey health care professionals working in private and public Austrian health-care organizations (hospitals, nursing homes, rehabilitation centers and sanatoriums). The questionnaire consisted of items from well-established scales. The collected data (*n* = 458) was analyzed using correlation and regression analyzes.

**Findings:**

Findings indicated that ethical leadership is significantly positively related to job satisfaction (*r* = 0.485, *p* < 0.01) and affective commitment (*r* = 0.461, *p* < 0.01) and is significantly negatively related to burnout (*r* = −0.347, *p* < 0.01). The results also suggest that frustration tolerance (ß = 0.101, *p* < 0.1) and emotional stability (ß = 0.093, *p* < 0.1) moderate the relationship between ethical leadership and burnout. Furthermore, a moderation effect of emotional stability in the ethical leadership and affective commitment relation was indicated. No moderation effect was found for frustration tolerance or emotional stability for the relationship between ethical leadership and job satisfaction.

**Practical implications:**

Ethical leadership emphasizes the socio-emotional dimension in a leader-employee relationship, which can easily be neglected in times of staff cuts and work overload. Leadership training should include the development of skills in how to visibly act as a moral person, as well as how to set clear ethical standards and communicate them to employees.

**Originality/value:**

This study adds value to the limited evidence on the beneficial role of ethical leadership in health care settings. In addition, frustration tolerance and emotional stability have not before been investigated as moderators.

## Introduction

Health-care organizations are expected to assume responsibility for society, promoting public health, respecting the rights and dignity of patients and ensuring humanity and patient safety. Ethics scandals and moral debates have triggered a rising concern about the ethics, integrity and social responsibility of health care organizations. The ethical behavior of organizations throughout different branches and sectors has increasingly gained attention in social scientific research, particularly in the leadership field (Brown and Treviño, 2006; Kalshoven *et al.*, 2011). Ethical leadership highlights the role of leaders in generating an atmosphere of trust, integrity and fairness (Mendonca and Kanungo, 2007). Given their positions of authority and status in the organizational hierarchy, leaders are receiving particular attention from employees (Neubert *et al.*, 2009). Their legitimate power, control of resources and responsibility enables them to influence both the ethical organizational climate and the individual behaviors of organizational members (Mayer *et al.*, 2009; Schaubroeck *et al.*, 2012).

By the beginning of this century, ethical leadership had been evolved as a distinct leadership construct. Brown *et al.* (2005) defined ethical leadership as “the demonstration of normatively appropriate conduct through personal actions and interpersonal relationships and the promotion of such conduct to followers through two-way communication, reinforcement and decision-making.” This definition unfolds two dimensions of ethical leadership: the moral person and the moral manager. First, the moral person dimension refers to the leader’s personal traits and behaviors. Ethical leaders are described as outstanding role models, altruistic, honest, trustworthy and fair individuals who show true concern and support for their subordinates, as well as care about their broader society (Treviño *et al.*, 2000). Second, the moral manager dimension refers to leaders’ role in shaping the behavior of others. Ethical leaders serve as role models for ethical conduct. Additionally, they actively model ethical behavior and encourage subordinates to act fairly and ethically through communicating clear ethical standards and values. Further, they enforce expected behaviors by using a reward system to guide desirable and undesirable conduct (Den Hartog and De Hoogh, 2009; Kalshoven *et al.*, 2011; Treviño *et al.*, 2000).

Other leadership theories, in particular transformational, authentic and servant leadership, also address the ethical dimension of leadership. These leadership theories include traits and ethical behaviors that refer to the moral person aspect of ethical leadership. In emphasizing leaders’ proactive influence on the ethical conduct of followers, the ethical leadership approach can be distinguished from other related theories (Brown and Treviño, 2006). Ethical leaders not only emphasize the importance of ethics through explicit communication of ethical standards but also shape ethical behaviors of subordinates relying on transactional forms of leadership to discipline and reward (un)ethical behaviors (Brown *et al.*, 2005).

Empirical research suggests that leaders who regularly demonstrate ethically normative behavior may elicit positive effects on a number of individual and organizational outcomes (Lawton and Paez, 2015). Specifically, scholars have found a positive relationship with followers’ perceptions and attitudes such as satisfaction with their leaders, perceived leader effectiveness, trust in management and organizational commitment (Brown *et al.*, 2005; Den Hartog and De Hoogh, 2009; Kalshoven *et al.*, 2011). While there is strong empirical support for a positive impact of ethical leadership on employees’ attitudes, the underlying mechanisms, in particular, the intervening variables remain an important area of research.

This study aims to contribute in two ways to the existing research on ethical leadership. First, the study intends to provide empirical evidence on the impact of ethical leadership in health care to add to the generalizability of the construct by exploring its relationship with three important employee work attitudes: job satisfaction, affective commitment and burnout. Second, using a follower-centered approach, the study examines the moderating role of two employee psychological resources: frustration tolerance and emotional stability. To the best of the authors’ knowledge, the influence of these personal resources on the relationship between ethical leadership and employee work attitudes has not yet been analyzed. Empirical evidence of the impact of ethical leadership on employee work attitudes in health care settings is scarce. The few studies that exist have reported a positive relationship between ethical leadership and organizational identification (Islam *et al.*, 2019; Lee, 2010), organizational commitment (Good *et al.*, 2018) and group performance (Walumbwa *et al.*, 2012) among nurses. A study conducted in a private health care organization in Germany observed a positive effect of ethical leadership on job satisfaction moderated by co-worker emotional support (Gerpott and Hackl, 2015). Another study conducted with physicians reported a beneficial effect of ethical leadership on emotional exhaustion, representing one dimension of burnout (Okpozo *et al.*, 2017).

In view of the identified research gaps and based on the postulated hypotheses presented in the following section, a research model was developed and empirically tested. The results of the study have both theoretical and managerial implications, which are presented in the final chapter.

## Theoretical background and hypothesis development

In developing our hypotheses, we relied on the social exchange theory, assuming that ethical leaders may influence employees’ attitudes in particular through the socio-emotional dimension. Social exchange relationships emerge from interactions between leaders and subordinates and they are motivated by the mutual benefits derived from the exchanges (Blau, 1964; Hansen, 2011). When employees perceive that they are being treated fairly and with respect, they are expected to enter into high-quality relationships with their leaders (Mayer *et al.*, 2009; Hansen, 2011). According to the principles of reciprocity, employees feel obliged to reciprocate a leader’s supportive treatment by returning beneficial behaviors (Blau, 1964). Employees who perceive their leaders to be strong ethical leaders are, therefore, more likely to reciprocate beneficial treatment by showing better performance, stronger commitment and higher satisfaction (Gerstner and Day, 1997; Mayer *et al.*, 2009).

### Ethical leadership and job satisfaction

Job satisfaction is described as “a pleasurable emotional state resulting from a favorable appraisal of one’s job as achieving or facilitating one’s job value” (Locke, 1969). Building on the social exchange theory, we assume that the key behaviors of an ethical leader such as fairness, caring and concern for employees, should encourage positive responses by employees. Health-care staff working with ethical leaders experience an environment characterized by interpersonal fairness, honesty and genuine concern for others. Thus, employees are expected to develop high-quality relationships and ethical work norms and should be experiencing more positive affective states of satisfaction and happiness (Avey *et al.*, 2012). In fact, a study by Avey *et al.* (2012) reports that when employees evaluate their overall level of job satisfaction, they also include implicit expectations concerning their leaders’ ethical conduct.

Our assumptions are supported by empirical research confirming the positive effect of ethical leadership on job satisfaction in various organizational settings (Avey *et al.*, 2012; Neubert *et al.*, 2009; Okan and Akyüz, 2015; Ogunfowora, 2014; Tu *et al.*, 2017; Yang, 2014). Drawing on the arguments stated above, it is hypothesized that ethical leadership is positively related to the job satisfaction of health care professionals:

H1. Ethical leadership of direct supervisors is positively related to employee job satisfaction in health care settings.

### Ethical leadership and affective commitment

Affective commitment refers to “the emotional attachment to, identification with and involvement in the organization” (Meyer *et al.*, 2002). It represents one of the three distinct components of organizational commitment (Meyer and Allen, 1991). This study focuses on affective commitment as it captures the emotional dimension and is likely to develop through the social exchange in a leader-follower relationship. Ethical leaders shape the working environment by means of their personal actions and interpersonal relationships. Building on the lines of social exchanges, we assume that ethical leadership should work as a facilitator, making health care staff reciprocate with attitudes and behaviors valued by the organization. When health care employees experience positive relationships with their subordinates, including being treated fairly and with respect and receiving support when needed, this should trigger the social exchange process, strengthen emotional attachment and make them feel more committed to the organization’s goals and values. Findings confirmed that strong leader-member exchange relationships were associated with higher levels of affective commitment (Hassan *et al.*, 2013; Shore *et al.*, 2006).

Guided by theoretical assumptions and empirical evidence (Demirtas *et al.*, 2017; Hassan *et al.*, 2013; Loi *et al.*, 2015; Neubert *et al.*, 2009; Neves and Story, 2015; Walumbwa *et al.*, 2011) this study hypothesizes that ethical leadership is positively related to affective commitment in health care professionals:

H2. Ethical leadership of direct supervisors is positively related to employee affective commitment in health care settings.

### Ethical leadership and burnout

Burnout is considered a psychological strain resulting from chronic emotional and interpersonal stressors on the job (Maslach, 2003; Lee and Ashforth, 1996). It refers to:

[…] a syndrome which emanates from an individual’s perception of unmet needs and expectations. It is characterized by progressive disillusion, with related psychological and physical symptoms which diminish one’s self-esteem and develop gradually over a period of time (Gold and Roth, 1993).

Health-care professions are widely recognized as stressful occupations that have a high risk for burnout. Facing working conditions characterized by various pressures concerning job content, workload and social expectations coupled with perceptions of low control over patient outcomes, health care professionals work in an environment that is very likely to induce symptoms of burnout (Chi and Chi, 2014). Ethical leaders show interest in the well-being of their followers, listen to their concerns and provide support when needed. Thus, ethical leadership creates a positive and psychologically safe work environment for health care staff. Working in a fair and secure organizational environment and receiving support when needed should reduce perceived work-related stress. Social support, especially, is expected to reduce feelings of emotional exhaustion, one central dimension of burnout (Halbesleben, 2006; Lee and Ashforth, 1996). Further, through the ethical manager dimension, leaders engage in open communication with employees, clarifying expectations and responsibilities that should reduce employees’ uncertainty when performing their jobs (Demirtas and Akdogan, 2015; Zheng *et al.*, 2015). Reducing feelings of uncertainty in their jobs and behaviors should translate into lower levels of work-related stress, thus reducing the risk of burnout. Also, in high-quality relationships, employees feel safe to express their inner feelings, which also buffers them from experiencing burnout (Mo and Shi, 2017).

The arguments above are strengthened by empirical findings suggesting that ethical leadership has the potential to reduce emotional exhaustion (Chughtai *et al.*, 2015; Zheng *et al.*, 2015) and to protect employees from burnout (Mo and Shi, 2017; Okpozo *et al.*, 2017). Drawing on these lines, this study hypothesizes that ethical leadership in the health care sector reduces employee burnout:

H3. Ethical leadership of direct supervisors is negatively related to employee burnout in health care settings.

### Moderating role of emotional stability and frustration tolerance

Emotional stability is linked to a low level of neuroticism, representing one of the Big Five personality dimensions (Barrick and Mount, 1991). Emotionally stable individuals are able to adequately cope with feelings of anxiety, emotionality, irritation, discontent and anger (Barbaranelli and Caprara, 2000). The social exchange theory approach predicts a positive impact of ethical leadership on job satisfaction and affective commitment because employees develop high-quality interpersonal relationships that foster positive work experiences. We assume that emotional stability affects the nature and quality of leader-employee relationships, which, in turn, moderates the effect of ethical leadership on employee attitudes. Emotionally stable employees are expected to perceive more positive work experiences, including relationships with colleagues and subordinates, than emotionally unstable employees struggling with feelings of discontent and anger. Thus, they may more readily develop high-quality interpersonal relationships with their leaders and may better respond to the positive working climate ethical leaders create. We, therefore, expect that the positive influence of ethical leadership on job satisfaction and affective commitment will be stronger in employees with high emotional stability than in those with low emotional stability. Further, it would be more challenging for ethical leaders to create a secure working environment for emotionally unstable employees, who may be anxious and tense and likely experiencing more feelings of stress. The high ethical standards and intense relationships with ethical leaders may cause high pressure and overload for emotionally unstable employees. Thus, they may benefit less from ethical leadership than emotionally stable employees. We, therefore, expect that the negative relationship between ethical leadership and employee burnout will be weaker when employee emotional stability is lower.

As a second moderating variable, we introduce frustration tolerance into our theoretical model. Frustration tolerance is closely related to emotional stability and is defined as “the ability of an individual to tolerate a frustrating situation for a longer period of time without distorting the objective factors of the situation” (Rosenzweig, 1938). Employees with a low frustration tolerance tend to give up quickly when efforts fail or are blocked (Costa and McCrae, 1992). They struggle to deal with stress, delays, obstacles or any situation that makes them uncomfortable. Health-care staff often experience situations of work overload or time pressure. Furthermore, health care employees are not always able to bring relief to patients or save their lives. We assume that health care employees with high frustration tolerance will experience more positive job events, even in challenging times. They may more readily respond to leaders’ ethical leadership resulting in higher job satisfaction and affective commitment. Thus, we expect that the positive influence of ethical leadership on job satisfaction and affective commitment will be stronger in employees high on frustration tolerance than in those low on frustration tolerance. Furthermore, it could be more challenging for ethical leaders to shape a psychologically safe work environment for employees with low frustration tolerance, as they more frequently experience feelings of stress when their efforts fail or do not achieve desired goals. Thus, they may benefit less from ethical leadership than employees with high frustration tolerance. We, therefore, expect that the negative influence of ethical leadership on burnout will be weaker when employee frustration tolerance is lower.

The influence of employees’ psychological resources has received very little attention in research on ethical leadership. To contribute to this research gap, this study introduces frustration tolerance and emotional stability into the research model:

H4. Frustration tolerance moderates the relationship between ethical leadership of direct supervisors and employee job satisfaction, affective commitment and burnout in health care settings.

H4.1. The higher employee frustration tolerance, the stronger the positive influence of ethical leadership on job satisfaction.

H4.2. The higher employee frustration tolerance, the stronger the positive influence of ethical leadership on affective commitment.

H4.3. The lower employee frustration tolerance, the weaker the negative influence of ethical leadership on burnout.

H5. Emotional stability moderates the relationship between ethical leadership of direct supervisors and employee job satisfaction, affective commitment and burnout in health care settings.

H5.1. The higher employee emotional stability, the stronger the positive influence of ethical leadership on job satisfaction.

H5.2. The higher employee emotional stability, the stronger the positive influence of ethical leadership on affective commitment.

H5.3. The lower employee emotional stability, the weaker the negative influence of ethical leadership on burnout.

## Methodology

### Research model

**Figure 1.**
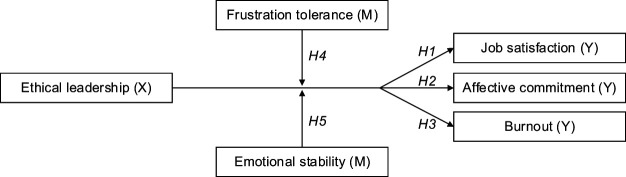
Research model

The above-mentioned hypotheses were schematized in a conceptual model (Figure 1) reflecting the relations between the independent (X), dependent (Y) and moderating variables (M).

### Data collection procedure

To test the conceptual model empirically, a questionnaire-based survey was conducted with health care professionals from different health care organizations (hospitals, nursing homes, rehabilitation centers and sanatoriums) located in two Austrian federal states (Kärnten and Salzburg). Two different modes of data collection were combined, involving web-based and paper-based modes. The online questionnaire, developed in Lime Survey 2.06+, was distributed via e-mail distribution lists of nine selected health care organizations and higher education institutions such as medical universities and schools of health and nursing. Educational institutions were involved to facilitate contacting graduates. This active sampling technique (Thielsch and Weltzin, 2012) ensured that only people contacted and invited via email were able to participate. Those who did not respond after eight weeks were sent a reminder. In addition, a print version of the questionnaire was distributed in the same facilities; thus, participation in the study also took place through self-selection. This mixed mode of data collection served to increase the size of the sample, which was important, as highly sensitive data was collected during the study and online surveys are known for their disadvantages (including issues related to anonymity, sampling frames, response rates and access to populations (Wright, 2005)). To assure the confidentiality of respondents and to reduce social desirability, detailed information was provided. During the data collection period of December 2016 to March 2017, 458 useable questionnaire responses were obtained. In line with general recommendations for determining sample size (Israel, 1992), thus obtained sample with a sampling error of ±5% and a confidence level of 0.95% was sufficient for representing the main population of approximately 200,000 people working in the Austrian health care sector.

### Study sample

The obtained sample (*n* = 458) consisted of 136 (29.7%) physicians, 175 (38.2%) nurses and 147 (32.1%) other health care professionals (midwives, physiotherapists, speech therapists, occupational therapists and radiology technologists). The majority of respondents were female (302 individuals, 65.9%) and between 25 and 44 years old (318 individuals, 69.5%). Of respondents, 317 (69.2%) worked in hospitals, 75 (16.4%) in nursing homes, 55 (12%) in rehabilitation centers and 11 (2.4%) in sanatoriums; 303 (66.2%) of these institutions were public. The majority of respondents (323 individuals, 70.5%) worked full time in an unlimited contractual relationship (344 individuals, 75.1%) and had been employed fewer than five years with their current organization (310 individuals, 67.7%).

### Measures

The questionnaire was composed of two sections. The first section included participants’ demographic information, including profession, age, gender, type of institution (hospital, nursing home, rehabilitation center or sanatorium), legal form of the institution (public/private), form of contract (limited/unlimited), extent of employment (full-time/part-time) and duration of employment. The second section consisted of items from existing measurement instruments that have been used in prior research and proved to be valid and reliable. Therefore, no new scales had to be developed. Most of the instruments were originally constructed in English. German versions of the scales that have been previously tested for validity and reliability were obtained.

To measure *ethical leadership*, a German version of the well-known 10-item ethical leadership scale (ELS) developed by Brown *et al.* (2005) was used. An example from the original scale is “My supervisor makes fair and balanced decisions.” Responses are scored on a five-point Likert scale in which 1 represents “never” and 5 represents “always.”

*Job satisfaction* was operationalized using the short version of the German 8-item Skala zur Messung von Arbeitszufriedenheit scale developed by Fischer and Lück (1972). An example statement is “I really enjoy the work.” Responses are scored on a five-point Likert scale, with answers ranging from “right” to “wrong.”

A German version of the 8-item affective commitment scale from Allen and Meyer (1990) was used to measure *affective commitment*. It includes statements like “I enjoy discussing my organization with people outside it” and “I really feel as if this organization’s problems are my own.” Responses are scored on a seven-point Likert scale ranging from “totally agree” to “totally disagree.”

*Burnout* was measured using nine items covering emotional exhaustion from the German version of the Maslach burnout inventory developed by Büssing and Prerrar (1992). Examples of statements include “I feel emotionally drained from my work” and “I feel frustrated by my job.” Responses are scored on a seven-point Likert scale ranging from “never” to “a few times a day.”

Six items from the Munich personality test created by Zerssen *et al.* (1988) were used to measure *frustration tolerance*. Examples of statements include “I get over disappointments quickly” and “I find it easy to relax.” Responses are scored on a four-point Likert scale ranging from “completely true” to “not true.”

*Emotional stability* was assessed using six items from the NEO-five factor inventory developed by Costa and McCrae (1992) and adapted into German by Borkenau and Ostendorf (1993). Items are arranged in the form of pairs of opposites (e.g. vulnerable – robust; helpless – self-confident) and are scored on a six-point Likert scale.

### Data analysis

Responses were coded so that high scores correspond to a high value of the construct of interest. Then, items on each scale were averaged within the scales to generate composite measures for each variable. To examine the reliability or accurateness and precision of the measurement instruments, a well-known test called Cronbach’s alpha was used. In general, reliability levels above 0.70 are considered satisfactory for research instruments (Tavakol and Dennick, 2011).

To test the proposed hypotheses, correlation and regression analyzes using IBM SPSS Statistics 25.0 were performed. To assess the predicted moderating effects of frustration tolerance and emotional stability, multiple linear regression models were conducted by considering the main effect of X, the main effect of M and the interaction effect X*M (Z) on Y. In the case of a statistically significant beta coefficient of the interaction term (Z), a moderating effect of M on the relationship between X and Y can be confirmed (Prado *et al.*, 2014). The moderation analyzes were further checked via models calculated with the PROCESS macro developed by Hayes (2018), in which the bootstrapping procedure is used and data for visualizing the interaction effects is provided.

## Results

### Reliability analysis

As shown in Table 1, the reliability status of measurement instruments was satisfactory with reliability levels above 0.779. That means that the scales used in the study accurately measured what they were intended to measure.

### Descriptive statistics and correlation analysis

Table 1 also presents values for mean, standard deviation and correlation among variables. As expected, ethical leadership was positively and significantly correlated with job satisfaction (*r* = 0.485, *p* < 0.01), affective commitment (*r* = 0.461, *p* < 0.01), frustration tolerance (*r* = 0.231, *p* < 0.01) and emotional stability (*r* = 0.130, *p* < 0.01) and was significantly negatively correlated with burnout (*r* = −0.347, *p* < 0.01). In examining the correlation matrix, no multicollinearity problems were identified, as correlation coefficients between the variables were below the recommended level of 0.80 (Bryman and Cramer, 1999).

### Hypotheses testing

As shown in Table 2, ethical leadership was found to be significantly and positively related to job satisfaction (ß = 0.485, *p* < 0.01) and affective commitment (ß = 0.461, *p* < 0.01) and significantly negatively related to burnout (ß = −0.347, *p* < 0.01). Thus, *H1, H2* and *H3* were supported.

**Figure 2.**
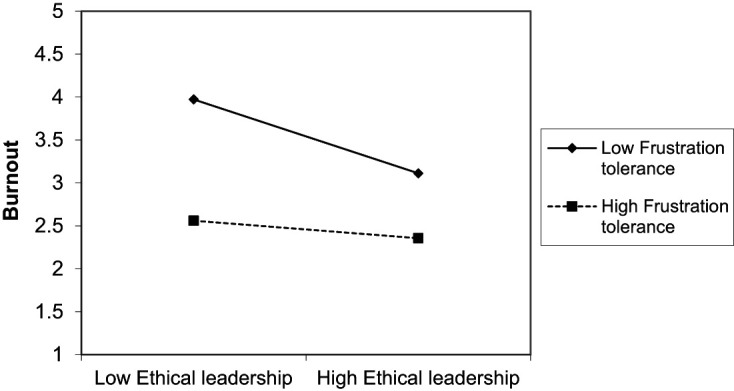
Moderation effect of frustration tolerance in the ethical leadership and burnout relation

**Figure 3.**
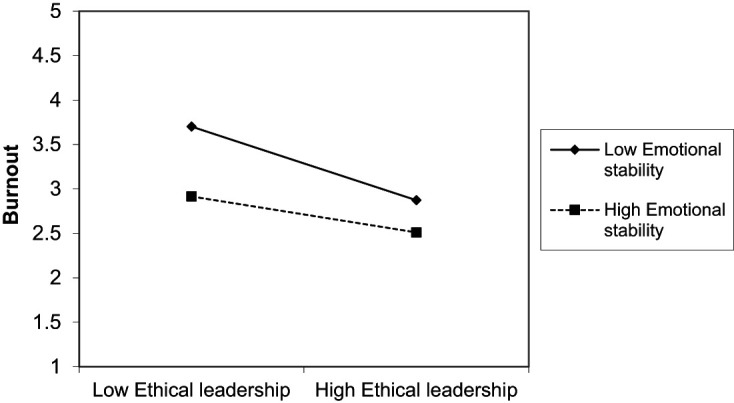
Moderation effect of emotional stability in the ethical leadership and burnout relation

Results suggest interactive effects of ethical leadership and employees’ psychological resources occurred. As displayed in Tables 3 and 4 and visualized in Figures 2 and 3, it was found that frustration tolerance (ß = 0.102, *p* < 0.05) and emotional stability (ß = 0.104, *p* < 0.05) significantly moderate the relationship between ethical leadership and burnout. The higher a person’s frustration tolerance or emotional stability, the weaker the negative influence of ethical leadership on burnout. Contrary to *H4.3*, the negative relationship of ethical leadership with burnout was weaker for employees high than for those low on frustration tolerance. Also, contrary to *H5.3*, the negative relationship of ethical leadership and burnout was weaker for employees high than for those low on emotional stability. Thus, *H4.3* and *H5.3* cannot be confirmed.

**Figure 4.**
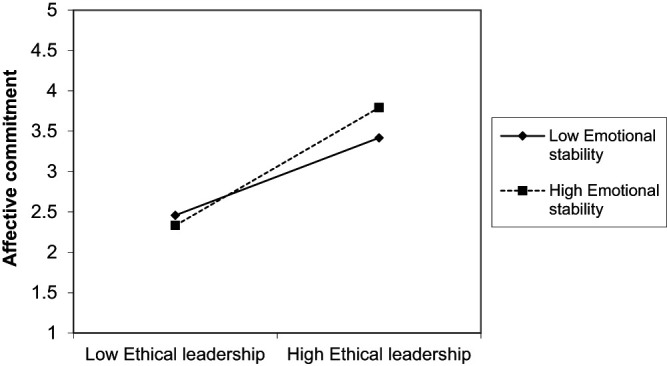
Moderation effect of emotional stability in the ethical leadership and affective commitment relation

Furthermore, as displayed in Tables 3 and 4, the study indicated a significant moderation effect of emotional stability in the relationship between ethical leadership and affective commitment (ß = 0.119, *p* < 0.01). As can be seen in Figure 4, the effects are close to one another at low ethical leadership and significantly higher at high ethical leadership; whereby high emotional stability additionally increases this effect. It can, therefore, be assumed that the higher a person’s emotional stability, the stronger the positive influence of ethical leadership on affective commitment. Thus, *H5.2* was supported. Indeed, no significant moderation effect of frustration tolerance could be found in the relationship between ethical leadership and affective commitment (ß = 0.003, ns). Thus, *H4.2* cannot be confirmed. Likewise, no significant moderation effect of frustration tolerance (ß = −0.028, ns) or emotional stability (ß = 0.023, ns) could be found in the relationship between ethical leadership and job satisfaction. Thus, our results also do not provide support for *H4.1* and *H5.1*.

### Control variables

Less significant findings were observed by controlling study results for demographic factors. It turned out that the control variables did not significantly contribute to the clarification of variance in job satisfaction (*R*^2^ = 0.035, *p* = 0.100), affective commitment (*R*^2^ = 0.025, *p* = 0.313) or burnout (*R*^2^ = 0.019, *p* = 0.517). The explanation of variance increased significantly only with the addition of ethical leadership. This means that the control variables did not significantly influence the research question of how ethical leadership affects job satisfaction, affective commitment and burnout. It could be shown only that the control variables alone significantly explain 4.2% (*p* = 0.043) of the variance in emotional stability and 4.5% (*p* = 0.026) of the variance in frustration tolerance, which represents a very small effect size. As shown in Table 3, hardly any significant results could be found by examining moderating relationships. It turned out only that affective commitment significantly increased with the length of employment in the moderating relationships. Aside from these results, the study showed that physicians rated their supervisors’ ethical leadership significantly lower than did other health professionals (ß = −0.170, *p* = 0.001).

## Discussion

The aim of the current research was twofold: First, to examine the impact of ethical leadership on employee work attitudes, including job satisfaction, affective commitment and burnout in the health care context and second, to investigate the moderating role of frustration tolerance and emotional stability in these relationships.

Consistent with previous studies conducted in different contexts, findings revealed a significant and positive direct effect of ethical leadership on job satisfaction and affective commitment and a significant negative direct effect on burnout. This means that leaders in health care organizations being perceived as strong ethical leaders by their employees can enhance employee job satisfaction and affective commitment and reduce the risk of burnout. Concerning the moderating role of frustration tolerance and emotional stability, results confirm this role in the relationship between ethical leadership and burnout. However, contrary to our assumptions, it turned out that the higher a person’s frustration tolerance or emotional stability, the weaker the negative influence of ethical leadership on burnout. A possible explanation for this could be that the safe and fair working environment ethical leaders can establish is especially relevant to those lower on emotional stability or frustration tolerance. Employees who are low on emotional stability or frustration tolerance tend to experience more feelings of stress and helplessness. This makes them likely to turn to their trustworthy leader, who shows true concern and support for them. There is less need for such leader support for emotionally stable or less frustrated employees. Therefore, the added “value” of ethical leadership in reducing stress and burnout is likely to be stronger for employees low on emotional stability or frustration tolerance. Furthermore, our study indicated a moderating role of emotional stability in the relationship between ethical leadership and affective commitment. As expected, it could be shown that the higher a person’s emotional stability, the higher the positive influence of ethical leadership on affective commitment. To the best of the authors’ knowledge, this issue has not been studied previously. Therefore, more studies are needed to check our results. Unlike what was hypothesized, no moderating effect of frustration tolerance in the relationship between ethical leadership and affective commitment was found. Likewise, no moderating effect of emotional stability or frustration tolerance was found in the relationship between ethical leadership and job satisfaction. A possible explanation could be that various contextual factors play an important role in shaping employee attitudes regarding job satisfaction and affective commitment, which were not considered in our model. For example, reward systems, work content and empowerment may also affect employee ratings on satisfaction and commitment. Next, our research supports previous studies, which identified a positive relationship between the duration of employment and employee commitment in the hospital setting (Kelarijani *et al.*, 2014; McNeese-Smith and Crook, 2003). Moreover, the results revealed differences in supervisors’ ratings between the professional groups. Physicians rated their supervisors’ ethical leadership significantly lower than did nurses and other health care professionals (midwives, physiotherapists, speech therapists, occupational therapists and radiology technologists). One explanation could be that physicians expect higher ethical standards from their superiors than do other professionals. Another explanation could be that nurses and other health care professionals like midwives or physiotherapists pay more attention to the socio-emotional relationship with their subordinates than do physicians. This difference in supervisor rating would be worth exploring deeper in future research studies.

### Theoretical implications

This study has enriched the current body of literature by providing several insights into the effect of ethical leadership in a health care context. In particular, the present study enriches leadership research by exploring the role of ethical leadership in reducing the risk of burnout. The findings provide additional evidence for the positive effect of ethical leadership. While numerous positive effects of ethical leadership have been documented by researchers, the conditions that alter the influence of ethical leadership are much less known. In taking a follower-centered approach, employees’ psychological resources were introduced as a possible moderator for the relationship between ethical leadership and employee work attitudes. Study results revealed a moderating effect of employee frustration tolerance and emotional stability in the relationship between ethical leadership and burnout, as well as a moderating effect of emotional stability in the relationship between ethical leadership and affective commitment. Finally, research findings contribute to the measurement of ethical leadership. The ELS exhibited very strong reliability in predicting ethical leadership in the health care context in Austria.

### Practical implications

Ethical leadership emphasizes the socio-emotional dimension in a leader-employee relationship, which can easily be neglected in times of time pressures and staff cuts. The results shed light on the importance of investing time in establishing a high-quality relationship with employees based on mutual trust and respect. From this perspective, ethical leadership does not refer only to the ethical conduct of a leader but covers a broader leadership range, including socio-emotional competencies like showing concern for employees, providing support when necessary, setting clear standards for teamwork and establishing a fair and safe work environment. Health-care leaders should always be aware of the consequences of their behaviors when treating employees fairly and honestly or not. Notwithstanding, the reader should keep in mind that ethical leadership emphasizes the relationship-oriented dimension of leadership and does not cover the full range of leadership tasks, especially those that refer to the task-related dimension of leadership.

Next, health care leaders should be aware of the importance of being role models for their employees. Health-care professionals often face situations involving ethical dilemmas or contexts in which the moral intensity of ethical decisions is high. Such situations draw the attention of employees to their leaders (Brown *et al.*, 2005; Brown and Treviño, 2006). They pay attention to the decision-makers to see how they handle the situation (Brown and Treviño, 2006). Such critical incidents shape employees’ perceptions of leadership behavior and will have a great influence on shaping their attitudes and ethical conduct (Brown *et al.*, 2005; Brown and Treviño, 2006). Moreover, the ethical leadership concept also encompasses assuming responsibility for the broader society (Cameron *et al.*, 2004). Paying attention to the ethical dimension of leadership would strengthen the social mission of a health care institution to provide a responsive environment for patients and their families and to engage in public health initiatives to improve the quality of life for all members of the community. Leadership training should include the development of socio-emotional skills to improve the leader-employee relationship and create a safe and fair working environment. Next, leaders should be trained in being role models for their employees. They should be provided with the necessary skills in how to effectively and visibly act as a moral person. Further, referring to the moral manager dimension, they should be trained in setting clear ethical standards and communicating their expectations to encourage compliance with ethical norms and morally appropriate behavior among organizational members.

### Limitations

Despite this study’s contributions toward a better understanding of the consequences and underlying mechanisms of ethical leadership, there are some limitations, which need to be mentioned. Although the study sample covered different health care organizations and various professional groups in health care, the sample was neither demographically diverse nor randomly selected. This limits the representativeness and generalizability of the results and application of these findings in other health care settings is needed. Second, only a few of the many possible variables were included in the research model. Empirical studies on ethical leadership have revealed a complex interplay between influencing factors and outcomes, which is difficult to capture in a single theoretical model. Furthermore, a common-method bias (Podsakoff *et al.*, 2003) produced by common source and measurement time cannot be excluded, especially, as the data for both the independent and dependent variables was obtained from the same respondents at one point in time. Single-source studies can result in distorted correlations between the variables investigated due to consistency motif, implicit theories and illusory correlations, leniency biases, social desirability or transient mood states (Podsakoff *et al.*, 2003). Therefore, similar studies in a health care context are needed to compare research findings.

### Future research

In view of the limitations outlined above, future researchers are encouraged to consider the following suggestions. First, ethical leadership is still at an early stage of conceptualization. Further research could be conducted with a larger sample and in other countries to review the results and to gain a better understanding of the mechanisms of ethical leadership in health care organizations. This is also an important requirement for the generalizability of these research results. Second, future researchers could use a multidimensional measure, for example, the ethical leadership at work questionnaire by Kalshoven *et al.* (2011), which distinguishes among seven ethical leader behaviors. A multi-dimensional measure should provide a clearer picture of how ethical leadership creates positive outcomes for employees and ultimately enhances the performance of health care organizations. Finally, future research should examine additional mechanisms by which ethical leadership influences employee attitudes. The present study investigated the moderating role of employee frustration tolerance and emotional stability on employee work attitudes. Future studies could explore other variables to gain more insight into the underlying network. For instance, the quality of interpersonal relationships or organizational settings like working climate may be variables that have an influence on the relationship between ethical leadership and employee work attitudes. In particular, future researchers are encouraged to consider the role of colleagues on ethical behavior in organizations. This field of study could be extended by including additional outcome variables, particularly crucial behavioral outcomes for health care organizations such as interactions with patients or compliance with the organization’s norms and guidelines.

## Figures and Tables

**Table 1.** Reliability analysis, descriptive statistics and correlation analysis

Variables	α	Mean	SD	1	2	3	4	5	6
Ethical leadership	0.908	3.128	0.867						
Job satisfaction	0.835	3.706	0.787	0.485**					
Affective commitment	0.809	4.134	1.162	0.461**	0.535**				
Burnout	0.893	2.158	0.842	−0.347**	−0.591**	−0.359**			
Frustration tolerance	0.779	2.630	0.560	0.231**	0.358**	0.125**	−0.431**		
Emotional stability	0.801	4.361	0.784	0.130**	0.240**	0.093*	−0.317**	0.533**	

Notes: **Correlation is significant at 0.01 (two-tailed); *Correlation is significant at 0.05 (two-tailed)

**Table 2.** Regression analyzes for testing *H1, H2* and *H3*

Hypotheses		*R*²	Adj. *R*²	β	*t*	*p*
*H1*	Ethical leadership → job satisfaction (+)	0.235	0.233	0.485	11.829	0.000
*H2*	Ethical leadership → affective commitment (+)	0.213	0.211	0.461	11.102	0.000
*H3*	Ethical leadership → burnout (−)	0.120	0.118	−0.347	−7.892	0.000

Note: Standardized betas are shown

**Table 3.** Regression analyzes for testing *H4* and *H5*

		Job satisfaction							
			*Model I*				*Model II*		
Hypothesis	Variable		β	*t*	*p*		β	*t*	*p*
*H4.1*	Ethical leadership		0.443	10.126	0.000		0.424	9.338	0.000
	Frustration tolerance		0.256	5.835	0.000		0.270	5.978	0.000
	EL × FT		−0.028	−0.651	0.516		−0.023	−0.523	0.601
	Gender						0.070	1.595	0.112
	Age						−0.086	−1.579	0.115
	Profession						−0.029	−0.534	0.594
	Extent of employment						0.007	0.160	0.873
	Form of contract						−0.004	−0.078	0.938
	Type of institution						0.065	1.277	0.202
	Legal form of institution						0.008	0.169	0.866
	Duration of employment						0.016	0.291	0.771
	*R*²	0.308				0.323			
	Adj. *R*^2^	0.302				0.303			
	Δ *R*^2^					0.016			
	F	56.195				16.120			
*H5.1*	Ethical leadership		0.472	10.763	0.000		0.452	9.969	0.000
	Emotional stability		0.197	4.442	0.000		0.215	4.736	0.000
	EL × ES		0.023	0.529	0.597		0.023	0.506	0.613
	Gender						0.060	1.332	0.184
	Age						−0.090	−1.623	0.105
	Profession						−0.026	−0.461	0.645
	Extent of employment						−0.011	−0.249	0.804
	Form of contract						−0.030	−0.623	0.534
	Type of institution						0.073	1.405	0.161
	Legal form of institution						0.002	0.051	0.959
	Duration of employment						0.013	0.234	0.815
	*R*²	0.280				0.298			
	Adj. *R*²	0.274				0.277			
	Δ *R*^2^					0.018			
	F	49.110				14.294			
		*Affective commitment*							
			*Model I*				*Model II*		
**H**	**Variable**		**β**	***t***	***p***		**β**	***t***	***p***
*H4.2*	Ethical leadership		0.463	9.920	0.000		0.471	9.760	0.000
	Frustration tolerance		−0.008	−0.173	0.862		0.007	0.138	0.890
	EL × FT		0.003	0.064	0.949		−0.004	−0.084	0.933
	Gender						0.070	1.483	0.139
	Age						−0.016	−0.280	0.780
	Profession						0.001	0.024	0.981
	Extent of employment						0.023	0.475	0.635
	Form of contract						−0.005	−0.095	0.924
	Type of institution						0.009	0.165	0.869
	Legal form of institution						0.019	0.374	0.709
	Duration of employment						0.133	2.291	0.023
	*R*²	0.213				0.235			
	Adj. *R*²	0.207				0.212			
	Δ *R*^2^					0.022			
	F	34.204				10.357			
*H5.2*	Ethical leadership		0.458	10.087	0.000		0.465	9.923	0.000
	Emotional stability		0.062	1.340	0.181		0.068	1.454	0.147
	EL × ES		0.119	2.614	0.009		0.112	2.409	0.016
	Gender						0.067	1.449	0.148
	Age						−0.018	−0.319	0.750
	Profession						0.018	0.322	0.747
	Extent of employment						0.011	0.221	0.825
	Form of contract						−0.013	−0.266	0.791
	Type of institution						−0.004	−0.076	0.940
	Legal form of institution						0.025	0.509	0.611
	Duration of employment						0.128	2.230	0.026
	*R*²	0.229				0.249			
	Adj. *R*²	0.223				0.227			
	Δ *R*^2^					0.020			
	F	37.462				11.190			
		*Burnout*							
			*Model I*				*Model II*		
**H**	**Variable**		**β**	***t***	***p***		**β**	***t***	***p***
*H4.3*	Ethical leadership		−0.243	−5.281	0.000		−0.232	−4.883	0.000
	Frustration tolerance		−0.358	−7.757	0.000		−0.379	−8.000	0.000
	EL × FT		0.102	2.258	0.024		0.102	2.240	0.026
	Gender						−0.056	−1.205	0.229
	Age						0.059	1.034	0.302
	Profession						−0.052	−0.923	0.357
	Extent of employment						0.019	0.408	0.683
	Form of contract						−0.069	−1.369	0.172
	Type of institution						−0.010	−0.190	0.850
	Legal form of institution						−0.031	−0.626	0.532
	Duration of employment						−0.074	−1.295	0.196
	*R*²	0.236				0.259			
	Adj. *R*²	0.230				0.237			
	Δ *R*^2^					0.023			
	F	39.084				11.777			
*H5.3*	Ethical leadership		−0.277	−5.930	0.000		−0.266	−5.497	0.000
	Emotional stability		−0.258	−5.456	0.000		−0.272	−5.601	0.000
	EL × ES		0.104	2.203	0.028		0.106	2.203	0.028
	Gender						−0.046	−0.959	0.338
	Age						0.075	1.265	0.207
	Profession						−0.044	−0.735	0.463
	Extent of employment						0.028	0.573	0.567
	Form of contract						−0.037	−0.717	0.474
	Type of institution						−0.027	−0.493	0.622
	Legal form of institution						−0.018	−0.345	0.730
	Duration of employment						−0.074	−1.251	0.212
	*R*²	0.180				0.197			
	Adj. *R*²	0.173				0.174			
	Δ *R*^2^					0.018			
	F	27.647				8.294			

Notes: H = Hypothesis; EL = Ethical leadership; FT = Frustration tolerance; ES = Emotional stability; standardized betas are shown

**Table 4.** Conditional effects of the focal predictor at the value of the moderator(s)

Hypothesis		*Burnout*					
*H4.3*	Frustration tolerance	Effect	se	*t*	*p*	LLCI	ULCI
	−0.630	−0.369	0.062	−5.979	0.000	−0.490	−0.248
	0.036	−0.260	0.040	−6.440	0.000	−0.340	−0.181
	0.536	−0.178	0.050	−3.534	0.000	−0.278	−0.079
*H5.3*	Emotional stability						
	−0.694	−0.381	0.055	−6.979	0.000	−0.488	−0.274
	−0.027	−0.310	0.041	−7.533	0.000	−0.391	−0.229
	0.806	−0.223	0.055	−4.078	0.000	−0.330	−0.115
		*Affective commitment*					
*H5.2*	Emotional stability	Effect	se	*t*	*p*	LLCI	ULCI
	−0.694	0.518	0.074	6.962	0.000	0.372	0.665
	−0.027	0.601	0.056	10.693	0.000	0.491	0.712
	0.806	0.705	0.074	9.471	0.000	0.559	0.852

Notes: H = Hypothesis; values of the moderators are the 16th, 50th and 84th percentiles
